# A biosystems approach to identify the molecular signaling mechanisms of TMEM30A during tumor migration

**DOI:** 10.1371/journal.pone.0179900

**Published:** 2017-06-22

**Authors:** Jiao Wang, Qian Wang, Dongfang Lu, Fangfang Zhou, Dong Wang, Ruili Feng, Kai Wang, Robert Molday, Jiang Xie, Tieqiao Wen

**Affiliations:** 1 Laboratory of Molecular Neural Biology, School of Life Sciences, Shanghai University, Shanghai, China; 2 School of Computer Engineering and Science, Shanghai University, Shanghai, China; 3 Shanghai Key Laboratory of Molecular Andrology, Institute of Biochemistry and Cell Biology, Shanghai Institute of Biological Science, Chinese Academy of Sciences, Shanghai, China; 4 Department of Biochemistry and Molecular Biology, University of British Columbia, Vancouver, Canada; University of South Alabama Mitchell Cancer Institute, UNITED STATES

## Abstract

Understanding the molecular mechanisms underlying cell migration, which plays an important role in tumor growth and progression, is critical for the development of novel tumor therapeutics. Overexpression of transmembrane protein 30A (TMEM30A) has been shown to initiate tumor cell migration, however, the molecular mechanisms through which this takes place have not yet been reported. Thus, we propose the integration of computational and experimental approaches by first predicting potential signaling networks regulated by TMEM30A using a) computational biology methods, b) our previous mass spectrometry results of the TMEM30A complex in mouse tissue, and c) a number of migration-related genes manually collected from the literature, and subsequently performing molecular biology experiments including the *in vitro* scratch assay and real-time quantitative polymerase chain reaction (qPCR) to validate the reliability of the predicted network. The results verify that the genes identified in the computational signaling network are indeed regulated by TMEM30A during cell migration, indicating the effectiveness of our proposed method and shedding light on the regulatory mechanisms underlying tumor migration, which facilitates the understanding of the molecular basis of tumor invasion.

## Introduction

Migration and invasion are key behaviors that distinguish benign from malignant tumors, enabling cell metastasis across tissue boundaries from the primary tumor location to a distant secondary site [1], thereby increasing disease severity and therapeutic challenges. Great effort, therefore, has been exerted to elucidate the mechanisms underlying cell migration and invasion [2–4]. Numerous studies have confirmed that invasive carcinoma cells acquire a migratory phenotype associated with various molecular and cellular mechanisms involved in cancer cell invasion [5, 6]. Tumor cell invasion is initiated by the loss of the cell-cell adhesion capacity from the primary tumor mass, and subsequent, changes in cell-matrix interactions enable the cells to invade surrounding tissue [4, 7]. Pathology observations *in vivo* indicate that tumor cells migrate in two main ways: individually and collectively [4, 8], knowledge of which has attracted efforts focusing on cell adhesion, epithelial to mesenchymal transition, angiogenesis, lymphangiogenesis and organ-specific metastasis [7]. Moreover, related molecules including cell adhesion factors [9, 10], growth factors [11], microRNA [3, 12], and lncRNA [13, 14] has been reported to be active during the metastatic cascade. Mounting evidence also indicates that inaddition to internal molecules, external influences modulate tumor migration and invasion [1, 2, 7, 15], such as chemical signals [16] and excessive amounts of exosomes released by tumor cells [17]. Nevertheless, the elaborate and complex regulatory mechanisms involved in the control of tumor cell migration remain unclear [1, 2, 7, 18].

TMEM30A is a terminally-glycosylated membrane protein that is ubiquitously expressed in mouse tissue [19]. The TMEM30A phospholipid flippase complex is known to play a role in cell migration [20] via the formation of membrane ruffles as a result of phospholipid translocation. However, it remains to be determined which molecules within the migratory machinery coordinate functions with this complex. In the present study, we aim to explore the molecular signaling mechanisms of TMEM30A using a biosystems approach to identify the signaling networks involved in tumor migration (S1 Fig). Our proposed method integrates computational and experimental approaches by first predicting potential signaling networks regulated by TMEM30A using computational biology methods, and subsequently performing molecular biology experiments including the *in vitro* scratch assay and qPCR. We assume that, in order to affect tumor migration, TMEM30A must regulate the expression of migration-related genes through certain pathways. The migration signaling network was constructed based on the STRING database together with our previous mass spectrometry results of the TMEM30A complex in mouse tissue and a number of migration-related genes manually collected from the literature. Subsequently, using both published data and our experimental results, the genes in the computationally-identified signaling network were validated and indeed shown to be regulated by TMEM30A during tumor migration, laying the foundation for the development of a potential cancer therapy.

## Materials and methods

### Computational prediction of the molecular mechanisms involved in migration

To identify signaling pathways regulated by TMEM30A during tumor migration, a number of migration-related genes were first manually collected from the literature. Subsequently, we obtained the protein-protein interaction network (PPIN) for *Mus musculus* from the STRING 10.0 database containing all known and predicted protein-protein interactions. In this version, there are 22668 distinct protein-encoding genes and 5109107 interactions, with weights between 1 and 999. The greater the weight, the higher the confidence, and the stronger the interaction. Subsequently, our previous mass spectrometry results of the TMEM30A complex in mouse tissue and the known migration-related genes from the literature were superimposed onto the PPIN from the STRING database. The sub-network of these selected proteins were extracted from the whole PPIN in STRING, which includes 377 proteins and 14161 interactions. DAVID was used to conduct the function and pathway enrichment analysis and the signaling network was visualized with Cytoscape [21]. http://dx.doi.org/10.17504/protocols.io.iapcadn [PROTOCOL DOI]

### Cell culture

Human hepatocellular carcinoma, SMMC-7721, and cervical adenocarcinoma, HeLa, cell lines were obtained from the Cell Bank of the Chinese Academy of Sciences (Shanghai, China). The cells were cultured in RPMI 1640 medium (Gibco, USA) supplemented with 10% fetal bovine serum (Hyclone, USA) and 1% penicillin/streptomycin (Gibco, USA). Maintenance was carried out under 5% CO_2_, in a 95% humidified atmosphere at 37°C. When the cells reached approximately 90% confluency, they were detached with 0.1% trypsin—ethylenediamine tetraacetic acid (Gibco, USA), seeded onto appropriate dishes, and incubated overnight. http://dx.doi.org/10.17504/protocols.io.iaqcadw [PROTOCOL DOI]

### Gene transfection

Transfection was carried out using *Lipofectamine*^™^ 2000 transfection reagent (Invitrogen, 12566014), according to the protocol provided. Briefly, cells were seeded in 24-well plates at a density of 1×10^5^ cells/500 μl and cultured overnight. 0.5 μg pcDNA3-ATP11A and 0.5 μg pcDNA3-TMEM30A plasmids in *Lipofectamine*^™^ 2000-RPMI 1640 medium (W/V, 1:1) (50 μl) were added dropwise to the cells, which were subsequently incubated under 5% CO_2_ in a 95% humidified atmosphere at 37°C for 6 h, following which the medium was replaced with fresh. http://dx.doi.org/10.17504/protocols.io.iarcad6 [PROTOCOL DOI]

### Measurement of cell migration

Cells were seeded at a density of 1×10^5^ cells/well in a 24-well plate and cultured under 5% CO_2_ at 37°C for 24 h. Following 6 h transfection, changes in the rate of cell migration were measured using a wound healing assay. Briefly, cells in each well were separated by a scratch-wound, a standardized scratch made with a P-20 pipette tip, and cells were observed every 12 h for 48 h using a Nikon Ti-S fluorescence microscope. The data were analyzed using the Image-Pro Plus software. http://dx.doi.org/10.17504/protocols.io.iascaee [PROTOCOL DOI]

### Total RNA extraction, cDNA synthesis, and qPCR

Cells were cultured in 24-well plates. 48 h post-transfection, the total cellular RNA was extracted using a Total RNA Extraction Kit (Promega, USA), according to the manufacturer’s protocol. The concentration of RNA was determined by measuring the absorbance at 260 nm, and 2 μg RNA was used for cDNA synthesis using an RT Master Mix (TaKaRa, Japan). qPCR amplification was performed using a mixture of Top Green qPCR Super Mix (Transgen, China), cDNA samples, and designated primers (Table 1). Relative gene expression was calculated by comparison of the CT value of the gene of interest with that of GAPDH, the internal control. http://dx.doi.org/10.17504/protocols.io.iaucaew [PROTOCOL DOI]

**Table 1:** List of primers used in qPCR.

Gene name	Primer sequence (5’ to 3’)
*GAPDH*	Upstream: TCACCACCATGGAGAAGGC
	Downstream: GCTAAGCAGTTGGTGGTGCA
*SRC*	Upstream: GAACCCGAGAGGGACCTTC
	Downstream: GAGGCAGTAGGCACCTTTTGT
*CDC42*	Upstream: CCATCGGAATATGTACCGACTG
	Downstream: CTCAGCGGTCGTAATCTGTCA
*WASL*	Upstream: CCCCAAATGGTCCTAATCTACCC
	Downstream: TGGAAATTGCTTGGTGTTCCTAT
*RHO*	Upstream: GGAAAGCAGGTAGAGTTGGCT
	Downstream: GGCTGTCGATGGAAAAACACAT
*SUB1*	Upstream: GAAGGTGAAATGAAACCAGGAAG
	Downstream: ACAGCTTTCTTACTGCGTCATC
*SLC2A1*	Upstream: GGCCAAGAGTGTGCTAAAGAA
	Downstream: CGATACCGGAGCCAATGGT
*CTNNB1*	Upstream: TGATGGAGTTGGACATGGCCATGGA
	Downstream: TGGCACCAGAATGGATTCCAGA
*ACTB*	Upstream: GTGACGTTGACATCCGTAAAGA
	Downstream: ATGAAGATCAAGATCATTGCTCCT
*HSP90B1*	Upstream: CAGAGAGAGGAAGAAGCTATTCAG
	Downstream: TTAAAAACTCGCTTGTCCCAGAT
*CLTC*	Upstream: AATGAAGGCCCATACCATGACT
	Downstream: TTATCCGTAACAAGAGCAACCG

### Statistical analysis

All data were analyzed using the GraphPad Prism software and are presented as the mean ± SEM. Average gaps in the cell migration assay were measured using the Image-Pro Plus software, and the rate of cell migration was analyzed by a two-way ANOVA. The mRNA level was analyzed by a one-way ANOVA. *P* < 0.05 is considered statistically significant.

## Results

### Construction of the molecular signaling networks involved in the TMEM30A complex

**Fig 1:**
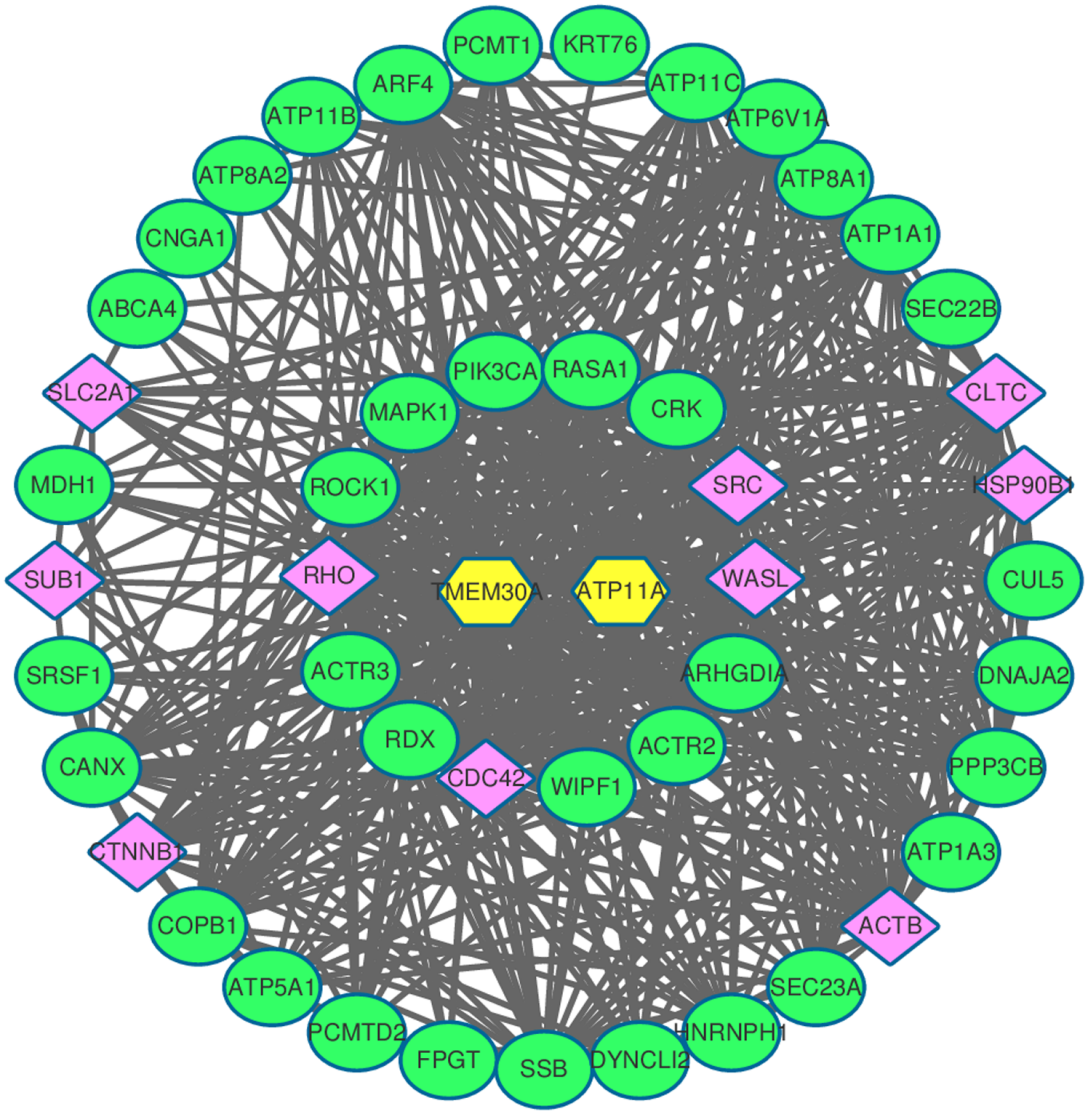
The migration-related signaling network regulated by TMEM30A. The largest connected components in the PPIN consisting of 347 genes expressed during migration and 14161 interactions. The first neighbors of TMEM30A were selected and a small network including 49 genes and 493 interactions was formed. The inner circles denote migration-related genes manually collected from the literature. The outer circles denote the genes predicted to interact with TMEM30A. The purple nodes were randomly-selected for experimental validation by qPCR.

In order to identify the molecular signaling networks involved in TMEM30A, our previous mass spectrometry results of the TMEM30A complex in mouse tissue and the known migration-related genes from the literature were superimposed onto the PPIN from the STRING database, since PPIN is widely used for the identification of signal transduction pathways. The largest connected component in the PPIN, consisting of 347 migration-related genes and 14161 interactions, was constructed for further analysis. To elucidate the most-related genes, the first neighbors of TMEM30A were selected, which formed a small network of 49 genes with 493 interactions (Fig 1). Moreover, migration-related genes were manually collected from the literature, of which 14 are found in the above PPIN (Table 2).

**Table 2:** The 14 known migration-related genes.

Gene symbol	Description	References (PIMID)
*PIK3CA*	Phosphatidylinositol 3-kinase, catalytic, alpha polypeptide	22064833; 27465249; 26829882; 27489350; 27511117; 27001433; 26747178; 25958091; 27460294; 28123856
*RASA1*	RAS p21 protein activator 1	25520857; 27101583; 26747707; 25778421; 21768288; 14639529
*CRK*	V-crk avian sarcoma virus CT10 oncogene homolog	27974675; 27656905; 27703373; 27418680; 27242434; 27210447; 27133071; 27052191
*SRC*	Rous sarcoma oncogene	27226554; 26773066; 24127286; 25711940; 24197068; 25870166; 27698945; 26645362
*WASL*	Wiskott-Aldrich syndrome-like	25621495; 24480980; 26500649; 19760510; 26656091; 22084245
*ARHGDIA*	Rho GDP dissociation inhibitor (GDI) alpha	27841340; 27726098; 26761212; 26062653; 25961457; 24859471; 24240172; 23867502; 22960606; 22928040
*ACTR2*	ARP2 actin-related protein 2	26370503; 24918434; 23644663; 16051170; 16004967; 15894313; 15727206
*WIPF1*	WAS/WASL interacting protein family, member 1	27863429; 26563365; 25169059; 18755005; 22823953; 17949983; 17312144; 16546573
*CDC42*	Cell division cycle 42	26450120; 25160664; 26634649; 23861058; 23562274; 23727359; 26633832; 26926567
*RDX*	Radixin	26438043; 24464681; 22199287; 22179828; 23339187; 22445717; 20739942; 15212902
*ACTR3*	ARP3 actin-related protein 3	26370503; 25682201; 24385601; 22370639; 16051170; 28115943; 16004967
*RHO*	Rhodopsin	28182100; 28143875; 28143705; 28135625; 28130497; 28123576; 28119242; 28115390
*ROCK1*	Rho-associated coiled-coil containing protein kinase 1	28123850; 28123576; 28115943; 28112365; 28054559; 27881000; 27852062; 27841867; 27666391
*MAPK1*	Mitogen-activated protein kinase 1	26346167; 26955781; 26514298; 26968871; 26945151; 26117268; 23051912; 26443721; 25770213; 25655003

Investigation of the functions of these genes is shown in Fig 1. We found that phospholipid and proton transport are significantly enriched (18 out of 49 genes) in the network, demonstrating that these genes are likely related to cell migration. SLC2A1, for instance, is critical for the growth and proliferation of tumor cells, with high SLC2A1 expression being associated with increased migration and poor patient survival [22]. Moreover, knockdown of SEC23A, a GTPase-activating protein critical for protein trafficking, has been shown to increase the proliferation, migration, and invasion of colorectal cancer cells [23]. Table 3 lists the top 20 most enriched functions of the genes in the identified signaling network, in which the biological process category from Gene Ontology was considered. Further, the enriched pathways from the KEGG database were screened for the genes in our predicted signaling network (Table 4). Among the 20 enriched pathways were those involved in the regulation of the actin cytoskeleton, adherens junctions, and focal adhesion, which are already known to be related to migration. From both the functional and pathway enrichment analyses, it can be seen that our identified signaling network including TMEM30A is indeed related to tumor migration.

**Table 3:** The top 20 most enriched functions of the genes in the predicted signaling network regulated by TMEM30A, where the biological process from Gene Ontology was considered.

Function	*P*-value	Genes in the predicted signaling network
Transport	1.06×10^−6^	*SEC23A*, *ATP1A3*, *ATP11A*, *ATP1A1*, *ATP11C*, *ABCA4*, *CNGA1*, *ATP6V1A*, *SRSF7*, *COPB1*, *ARF4*, *SLC2A1*, *ATP8A2*, *SEC22B*, *ATP5A1*, *DYNC1I2*, *TMEM30A*, *ATP8A1*
Phospholipid transport	1.61×10^−6^	*ATP8A2*, *ATP11A*, *ATP11C*, *TMEM30A*, *ATP8A1*
Ephrin receptor signaling pathway	4.21×10^−6^	*CDC42*, *WASL*, *CRK*, *SRC*, *RASA1*
Spindle localization	6.30×10^−5^	*ACTR3*, *ACTR2*, *WASL*
ATP hydrolysis-coupled proton transport	7.97×10^−5^	*ATP6V1A*, *ATP1A3*, *ATP1A1*, *ATP5A1*
Lipid transport	1.80×10^−4^	*ATP8A2*, *ATP11A*, *ATP11C*, *TMEM30A*, *ATP8A1*
Adherens junction organization	2.25×10^−4^	*CDC42*, *SRC*, *CTNNB1*
Phospholipid translocation	3.43×10^−4^	*ATP11C*, *ABCA4*, *TMEM30A*
Actin filament organization	1.23×10^−3^	*ACTR3*, *ACTR2*, *CDC42*, *WASL*
Cellular response to platelet-derived growth factor stimulus	1.55×10^−3^	*RDX*, *SRC*, *RASA1*
Vesicle-mediated transport	2.07×10^−3^	*SEC23A*, *COPB1*, *ARF4*, *SEC22B*, *CLTC*
Establishment or maintenance of cell polarity	2.13×10^−3^	*ACTR3*, *ACTR2*, *CDC42*
Meiotic cytokinesis	5.08×10^−3^	*ACTR3*, *ACTR2*
Actin cytoskeleton organization	5.62×10^−3^	*ACTR2*, *ROCK1*, *WASL*, *WIPF1*
Rho protein signal transduction	6.10×10^−3^	*CDC42*, *ROCK1*, *ARHGDIA*
Asymmetric cell division	7.61×10^−3^	*ACTR3*, *ACTR2*
Positive regulation of phospholipid translocation	7.61×10^−3^	*ATP8A2*, *ATP8A1*
Meiotic chromosome movement towards spindle pole	7.61×10^−3^	*ACTR3*, *ACTR2*
Fc-gamma receptor signaling pathway involved in phagocytosis	1.01×10^−2^	*CDC42*, *WASL*
Response to drugs	1.07×10^−2^	*ATP1A3*, *ATP1A1*, *SRC*, *RASA1*, *CTNNB1*

**Table 4:** Pathway enrichment analysis of genes in the predicted signaling network regulated by TMEM30A, where the pathway information was obtained from the KEGG database.

Pathway	*P*-value	Genes in the predicted signaling network
Bacterial invasion of epithelial cells	2.83×10^−8^	*ACTB*, *CDC42*, *PIK3CA*, *WASL*, *CLTC*, *CRK*, *SRC*, *CTNNB1*
Thyroid hormone signaling pathway	3.73×10^−7^	*ACTB*, *MAPK1*, *SLC2A1*, *ATP1A3*, *PIK3CA*, *ATP1A1*, *SRC*, *CTNNB1*
Regulation of actin cytoskeleton	2.34×10^−6^	*ACTB*, *MAPK1*, *CDC42*, *ROCK1*, *PIK3CA*, *RDX*, *WASL*, *CRK*, *SRC*
Adherens junctions	1.19×10^−5^	*ACTB*, *MAPK1*, *CDC42*, *WASL*, *SRC*, *CTNNB1*
Salmonella infection	1.76×10^−5^	*ACTB*, *MAPK1*, *CDC42*, *ROCK1*, *WASL*, *DYNC1I2*
Proteoglycans in cancer	1.87×10^−5^	*ACTB*, *MAPK1*, *CDC42*, *ROCK1*, *PIK3CA*, *RDX*, *SRC*, *CTNNB1*
Focal adhesion	2.13×10^−5^	*ACTB*, *MAPK1*, *CDC42*, *ROCK1*, *PIK3CA*, *CRK*, *SRC*, *CTNNB1*
cGMP-PKG signaling pathway	7.29×10^−5^	*MAPK1*, *ROCK1*, *ATP1A3*, *PPP3CB*, *PIK3CA*, *ATP1A1*, *CNGA1*
VEGF signaling pathway	1.14×10^−4^	*MAPK1*, *CDC42*, *PPP3CB*, *PIK3CA*, *SRC*
Chemokine signaling pathway	1.55×10^−4^	*MAPK1*, *CDC42*, *ROCK1*, *PIK3CA*, *WASL*, *CRK*, *SRC*
Renal cell carcinoma	1.75×10^−4^	*MAPK1*, *CDC42*, *SLC2A1*, *PIK3CA*, *CRK*
Rap1 signaling pathway	2.50×10^−4^	*ACTB*, *MAPK1*, *CDC42*, *PIK3CA*, *CRK*, *SRC*, *CTNNB1*
Fc gamma R-mediated phagocytosis	4.20×10^−4^	*MAPK1*, *CDC42*, *PIK3CA*, *WASL*, *CRK*
Oxytocin signaling pathway	5.03×10^−4^	*ACTB*, *MAPK1*, *ROCK1*, *PPP3CB*, *PIK3CA*, *SRC*
Aldosterone-regulated sodium reabsorption	6.31×10^−4^	*MAPK1*, *ATP1A3*, *PIK3CA*, *ATP1A1*
Pathways in cancer	1.20×10^−3^	*MAPK1*, *CDC42*, *HSP90B1*, *ROCK1*, *SLC2A1*, *PIK3CA*, *CRK*, *CTNNB1*
cAMP signaling pathway	1.36×10^−3^	*MAPK1*, *ROCK1*, *ATP1A3*, *PIK3CA*, *ATP1A1*, *CNGA1*
Leukocyte transendothelial migration	1.65×10^−3^	*ACTB*, *CDC42*, *ROCK1*, *PIK3CA*, *CTNNB1*
Neurotrophin signaling pathway	1.70×10^−3^	*MAPK1*, *CDC42*, *PIK3CA*, *CRK*, *ARHGDIA*
Axon guidance	2.09×10^−3^	*MAPK1*, *CDC42*, *ROCK1*, *PPP3CB*, *RASA1*

### Identification of the overexpression of TMEM30A and ATP11A

**Fig 2:**
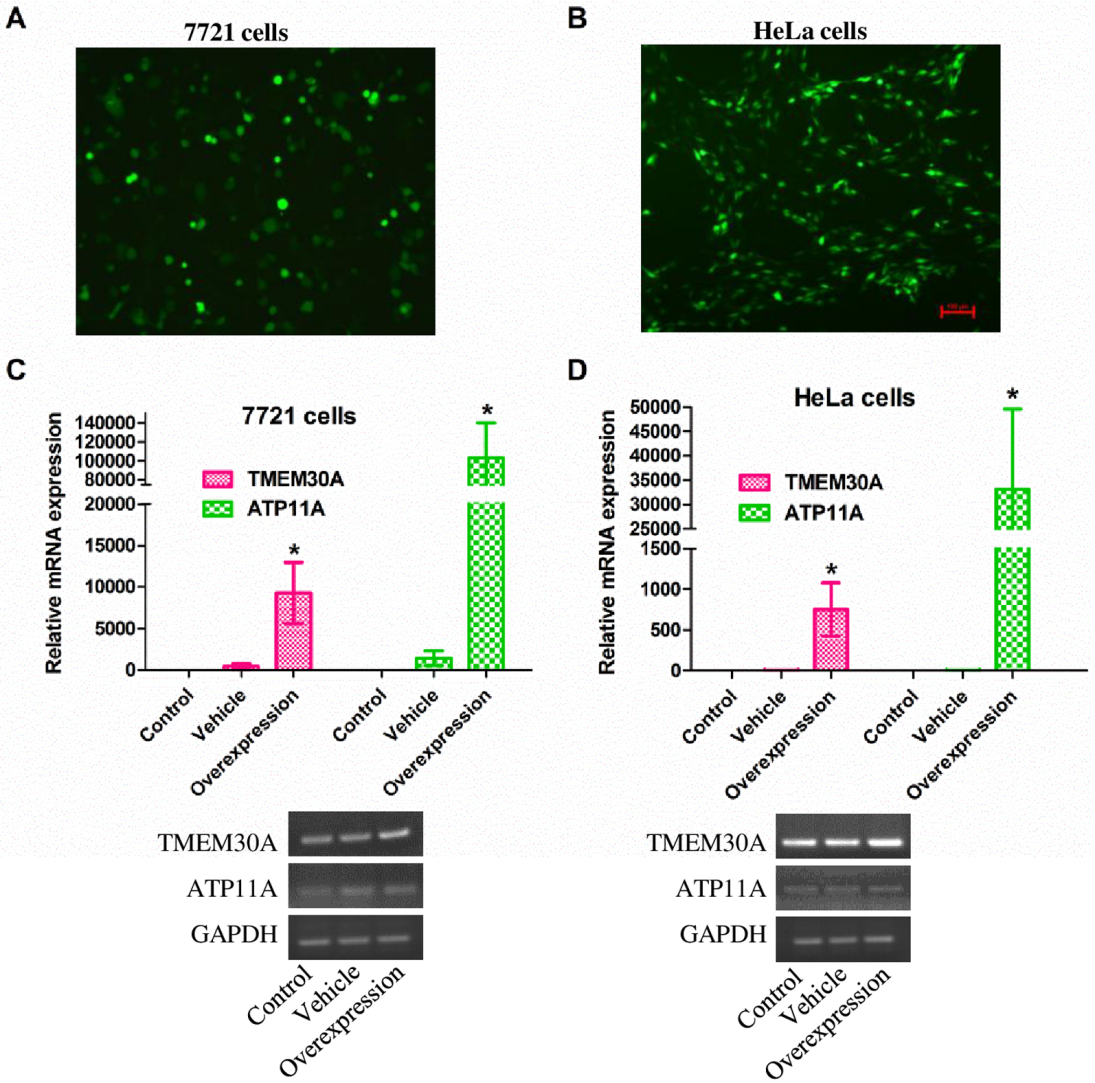
Identification of the overexpression of TMEM30A and ATP11A in 7721 or HeLa cells. (A and B) 7721 or HeLa cells were viewed under a Nikon fluorescence microscope 24 hours following transfection with vectors capable of expressing green fluorescent protein (GFP) fusion proteins. (C and D) The mRNA levels of TMEM30A and ATP11A were assessed by qPCR in 7721 or HeLa cells. The products of the qPCR were separated on 1.5% agarose gels and visualized using ethidium bromide staining. Abundance of each mRNA transcript is expressed relative to GAPDH as an internal control. Data are represented as the mean ± SEM. n = 4. *, *p* < 0.05; Overexpression vs. Vehicle.

To further verify that our predicted signaling network is indeed regulated by TMEM30A, we investigated its effects on several randomly selected genes by manipulating TMEM30A expression. TMEM30A structurally resembles the β-subunit of the P4-ATPase, which is abundant in our mass spectra (Fig 1), and is also responsible for the preservation of lipid gradients in mammalian tissues [24], thus ATP11A, a member of the P4-ATPase family, was selected since it has been reported to be a novel predictive marker for colorectal cancer prognosis [25]. TMEM30A and ATP11A were therefore overexpressed in two different human tumor cell lines, hepatocellular carcinoma SMMC-7721 and cervical adenocarcinoma HeLa. Green fluorescence images revealed that overexpression plasmids were efficiently transfected into 7721 (Fig 2A) and HeLa cells (Fig 2B), respectively. In the qPCR results, the mRNA expression levels of ATP11A and TMEM30A were significantly increased in both 7721 (Fig 2C) and HeLa cells (Fig 2D). These results indicated that TMEM30A and ATP11A were successfully overexpressed in these two cancer cell lines.

### Tumor migration mediated by TMEM30A

**Fig 3:**
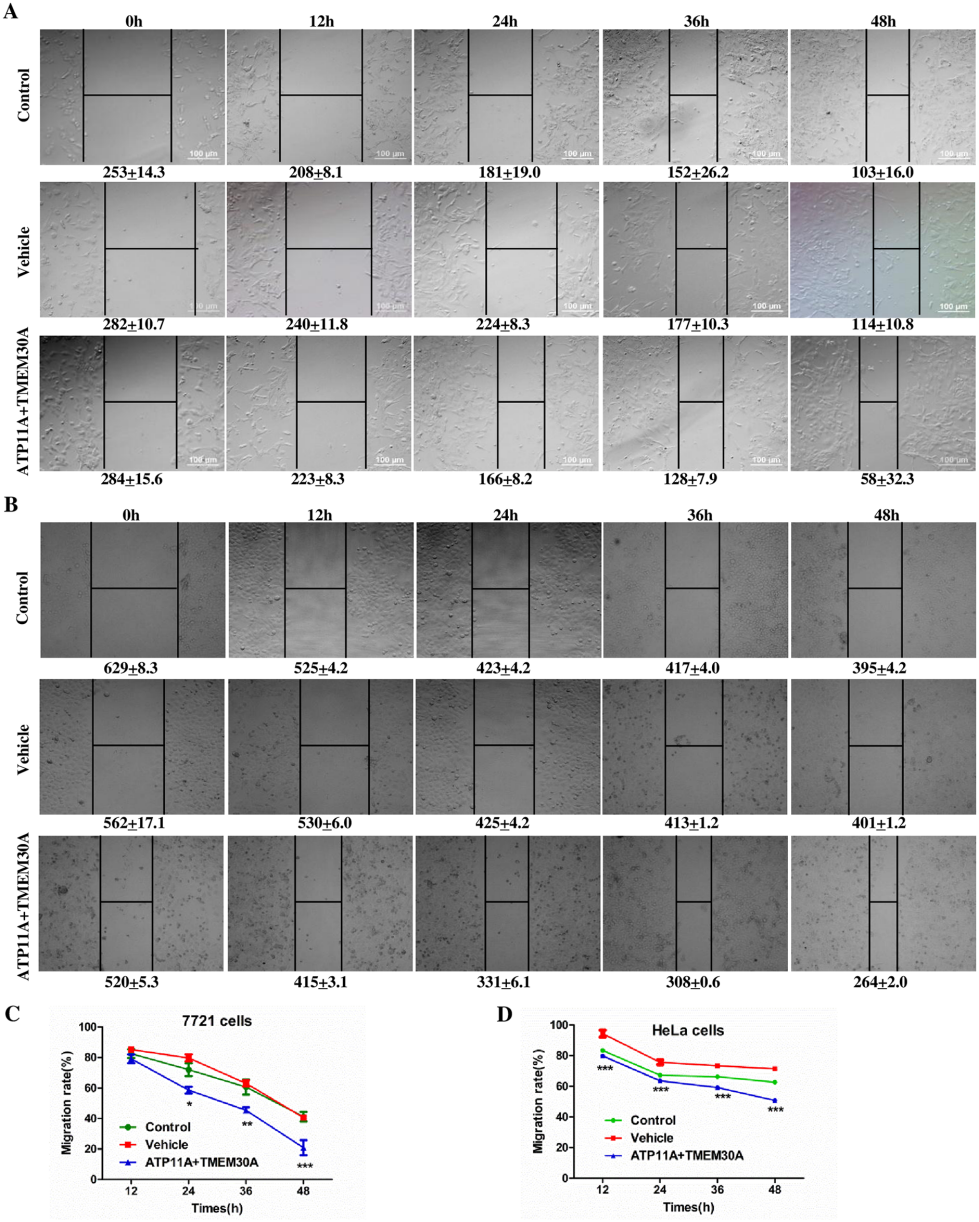
Effect of TMEM30A and ATP11A on 7721 or HeLa cell migration using an wound-healing assay. Representative images of the scratched areas in each condition at different time points were photographed. (A and B) Average gaps in three groups of 7721 or HeLa cells, respectively. (C and D) Statistical analysis of the migration rate in three groups of 7721 or HeLa cells, respectively. Data are represented as the mean ± SEM. 7721 cells, n = 6; HeLa cells, n = 3. *, *p* < 0.05; **, *p* < 0.01; ***, *p* < 0.001; ATP11A+TMEM30A vs. Vehicle.

A wound-healing assay was conducted to assess the effect of TMEM30A and ATP11A on 7721 or HeLa cell migration. Representative images of the scratched areas in each condition at different time points were photographed. The results showed that the scratched areas in both 7721 (Fig 3A) and HeLa (Fig 3B) cells decreased from 12 to 48 h. Moreover, statistical analysis revealed that the migration rate of 7721 cells was significantly increased from 24 h in the ATP11A+TMEM30A group (Fig 3C). Similarly, the migration rate of HeLa cells also dramatically increased from 12 h in the ATP11A+TMEM30A group (Fig 3D). Taken together, overexpression of TMEM30A and ATP11A led to a significant increase in the migration rate of both 7721 and HeLa cells, indicating that TMEM30A is involved in cell migration.

### Experimental validation of the signaling network

**Fig 4:**
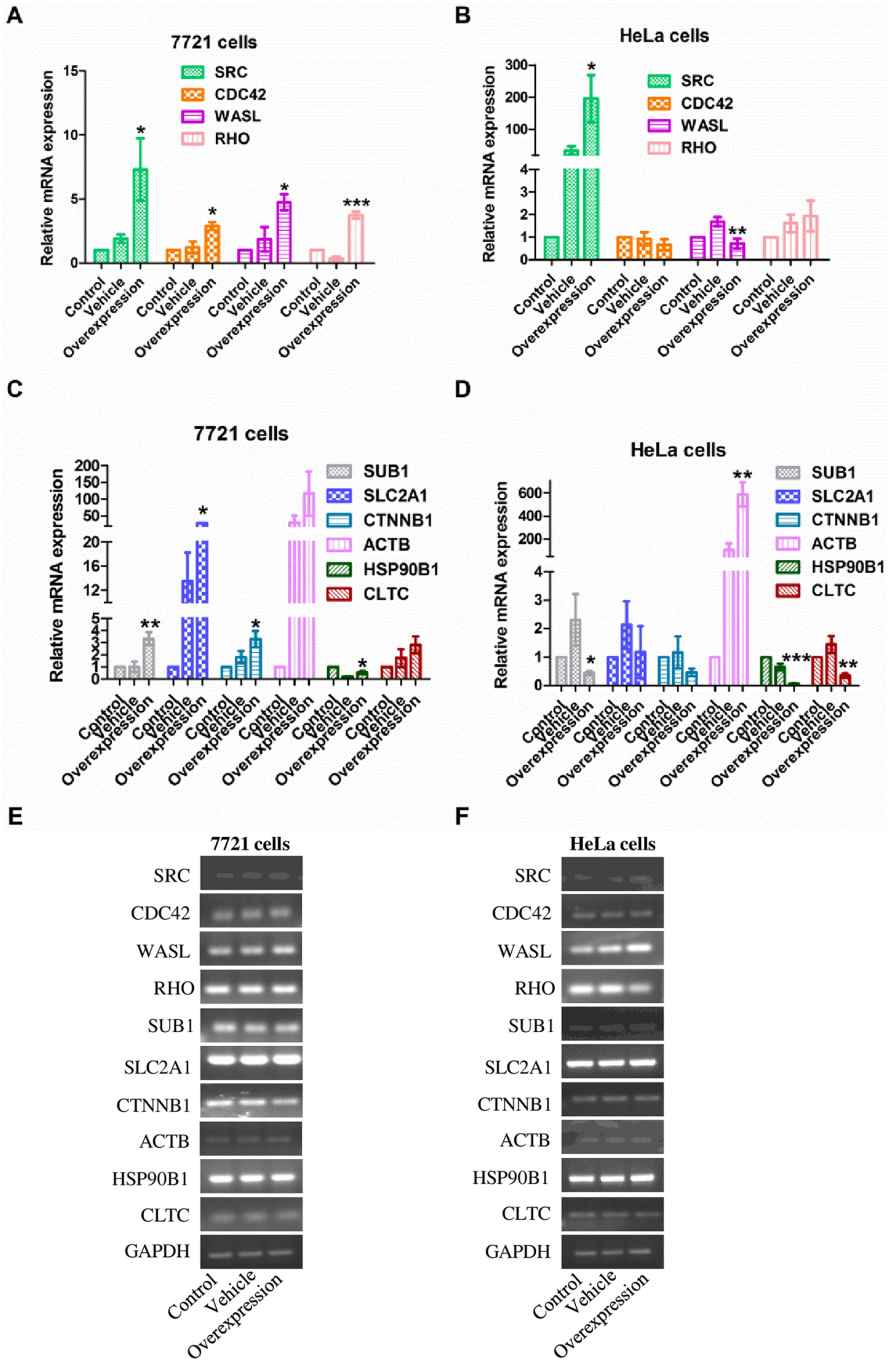
The expression of ten genes was evaluated by qPCR in 7721 or HeLa cells. The overexpression group represents the coexpressed TMEM30A and ATP11A. (A and B) The 4 genes collected from the literature were assessed by qPCR in the control group, vehicle group, and overexpression group, in 7721 or HeLa cells. (C and D) The 6 predicted genes were assessed by qPCR in the control group, vehicle group, and overexpression group, in 7721 or HeLa cells. (E and F) The products of the qPCR were separated on 1.5% agarose gels and visualized by ethidium bromide staining. Abundance of each mRNA transcript is expressed relative to GAPDH as an internal control. Data are represented as the mean ± SEM. n = 4. *, *p* < 0.05; **, *p* < 0.01; ***, *p* < 0.001; Overexpression vs. Vehicle.

As can be seen in Fig 4A and 4B, four genes, *SRC*, *CDC42*, *WASL*, and *RHO* were selected from 14 genes (Table 2) that had already been confirmed to be related to migration. The mRNA levels of *CDC42*, *WASL*, and *RHO* were significantly increased in 7721 cells. However, in HeLa cells, the mRNA expression of *SRC* was significantly increased, and that of *WASL* was dramatically decreased. As shown in Fig 4C and 4D, another 6 genes, *SUB1*, *SLC2A1*, *CTNNB1*, *ACTB*, *HSP90B1*, and *CLTC* were selected from the remaining unknown migration-related genes in order to confirm their relationship with cancer cell migration. In the present study, the results show that the mRNA levels of *SUB1*, *SLC2A1*, *CTNNB1*, *ACTB*, and *CLTC* were increased in 7721 cells but decreased in HeLa cells, with the exception of *ACTB*, which was also significantly increased. Moreover, the products of the qPCR were subjected to agarose gel electrophoresis to reflect the abundance of each mRNA transcript in 7721 (Fig 4E) or HeLa cells (Fig 4F), which were consistent with above results.

## Discussion

In recent years, many research groups have become interested in investigating pathways from protein-protein interaction and high-throughput data, with various computing methods having been developed to indicate molecular pathways [26–28]. In this work, we obtained the protein-protein interaction data for *Mus musculus* from the STRING database 10.0, which is an open source database for all known and predicted protein-protein interactions. Subsequently, the proteins from our previous TMEM30A complex mass spectrometry experiment in mouse tissue and a number of manually collected migration-related proteins were superimposed onto the PPIN to elucidate the signaling mechanisms of TMEM30A during tumor migration. Our results indicate that TMEM30A and ATP11A promote the migration of tumor cells (Fig 3), which is consistent with reports that depletion of either TMEM30A or ATP8A1 causes a severe defect in cell migration [20]. Taken together, it can be speculated that TMEM30A binds to P4-ATPase, which participates in the migration process of tumor cells. The enriched functions (Table 3) and pathways (Table 4) of our predicted signaling network, including regulation of the actin cytoskeleton, adherens junctions, and focal adhesion, are already known to be related to migration, which increases the credibility of our predicted network.

As indicated by our results, the mRNA expression levels of *SRC*, *CDC42*, *WASL*, and *RHO* were all changed during cancer cell migration (Fig 4A and 4B). *SRC* is a proto-oncogene that is aberrantly activated in most types of cancer [29], which is consistent with our results showing that *SRC* is significantly increased in both cell types. Further, the FAK-SRC signaling pathway regulates the invasion of lung cancer [30] and Ewing sarcoma cells [29], indicating that SRC may be a key therapeutic target for cancer treatment. It is widely accepted that all modes of migration require RHO GTPase, including RHO and CDC42 (Fig 4A and 4B) due to its effects on the regulation of actin dynamics that plays a critical role in cell migration depending on the environment [31, 32]. It has been reported that enhanced activity of RHO stimulates the *in vitro* migration of papillary thyroid cancer (PTC) [33], hepatocellular carcinoma [34] and human colon cancer cells [35]. CDC42 plays an important role in the establishment of cell migratory polarity and persistence via its activation in clear cell renal cell carcinoma (ccRCC) [36]. This is consistent with our results showing that upregulation of CDC42 and RHO promotes the migration of 7721 and HeLa cells. In addition, there was an expression change in WASL, an effector of the Rho protein, which was dramatically decreased in HeLa cells and increased in 7721 cells. WASL is a regulator of actin polymerization and is responsible for membrane ruffling [37]. The reverse expression of *WASL* in these two tumor cell lines implies a complicated function of *WASL* in different cancer types. According to the literature, knockdown of *WASL* significantly reduces the number of membrane protrusions and inhibits breast cancer cell invasiveness [38], whereas increased WASL promotes lymph node metastasis [38]. The same reverse expression was also found with *SUB1*, which suggests that further experiments are needed to clarify the involvement of these two genes in tumor migration.

Furthermore, the expression of *SLC2A1*, *CTNNB1*, *ACTB*, and *CLTC* were significantly changed in only one type of cancer during migration (Fig 4C and 4D). For instance, ACTB is a highly conserved protein well-known for its regulation of the actin cytoskeleton and adherens junctions, which are necessary for both normal and tumor cell migration [31, 39]. The significant change in the expression level of ACTB in HeLa cells indicates that ACTB may execute a special function in HeLa cell migration. In addition, SLC2A1 is a transporter that facilitates glucose entering the cytoplasm [40]. It is critical for the growth, proliferation, and migration of tumor cells, and its high expression is associated with hepatocellular carcinoma [40] and poor cancer survival [22]. Hepatocellular carcinoma cells were used in our experiments, with a consistent change in mRNA levels being observed by qPCR (Fig 4C). Moreover, CLTC, a membrane protein like TMEM30A and its partner P4-ATPase [24], is expressed in pancreatic cancer cells and tumor blood vessels [41], being involved in the ubiquitous uptake of a variety of macromolecules, membrane transporters, and adhesion molecules [41, 42]. According to our results, CLTC was dramatically decreased in HeLa cells following overexpression of TMEM30A, indicating involvement in HeLa cell migration, and implying that TMEM30A and its partner P4-ATPase, may function together with CLTC on the membrane to trigger downstream signaling pathways involved in tumor migration. The slight difference in the expression of these genes in different cancer cell types also suggests the delicate and complex regulatory mechanisms involved in tumor cell migration, implying that anti-cancer therapeutics are complicated and highly dependent on cell type. Interestingly, considering that the overexpression of TMEM30A promotes the migration of these two types of cancer cells, TMEM30A may be a novel therapeutic target in cancer treatment.

Proteins from the same signaling pathway are usually co-expressed, in addition, the results of our biological verification experiments showed that the mRNA levels of the selected genes changed along with increased expression of TMEM30A, therefore, novel signaling pathway could be predicted by differentially expressed genes from proteomic data [28]. Although the protein-protein interactions are undirected, signaling transduction is normally the process by which a signal is transmitted through the cell as a series of molecular events. Proteins responsible for detecting stimuli are generally located on the plasma membrane, and changes in protein phosphorylation state give rise to a cascade of biochemical events mediated by protein kinase, which ultimately regulate the function of transcription factors. Furthermore, many researchers have also developed various algorithms and tools to infer signaling pathways using the protein-protein interactions deposited in public database [26, 43, 44]. Thus, we could infer the direction that we verified experimentally to be in the following order: transmembrane protein (ATP11B, TMEM30A, CLTC, RHO), cytoplasmic protein (WASL, CDC42, ACTB, SEC23A, HSP90B1, CTNNB1), kinase (SRC), transcription factor activator (SUB1) (S2 Fig).

In conclusion, by combining the predictive power of computational biology and molecular biology experiments, we successfully identified the migration-related signaling network involved in the TMEM30A complex, and the randomly selected genes were subsequently validated and shown to indeed be regulated by TMEM30A during tumor migration. In particular, we found that TMEM30A regulates several migration-related genes including *SUB1*, *SLC2A1*, *CTNNB1*, *ACTB*, and *CLTC* during tumor migration, which further demonstrates the effectiveness of our proposed method. We believe that our identified signaling network can shed light on the regulatory mechanisms underlying tumor migration and facilitate the understanding of the molecular basis of tumor invasion.

## Supporting information

S1 Fig A flowchart of the molecular signaling networks identified to be involved in the TMEM30A complex during tumor migration by exploring the PPIN and known genes in the literature. (TIF)

S2 Fig The verified signaling pathway extracted from the whole PIN. (TIF)

S1 Table Primary data used in Fig 2. (XLSX)

S2 Table Primary data used in Fig 3. (XLSX)

S3 Table Primary data used in Fig 4. (XLSX)

S1 File Original and unadjusted gels for products identification after qPCR. (PPTX)
