# Supplementary figures and images for: A biosystems approach to identify the molecular signaling mechanisms of TMEM30A during tumor migration
Source: PLoS One. 2017 Jun 22;12(6):e0179900. doi: 10.1371/journal.pone.0179900 (PMC5481017; doi:10.1371/journal.pone.0179900)

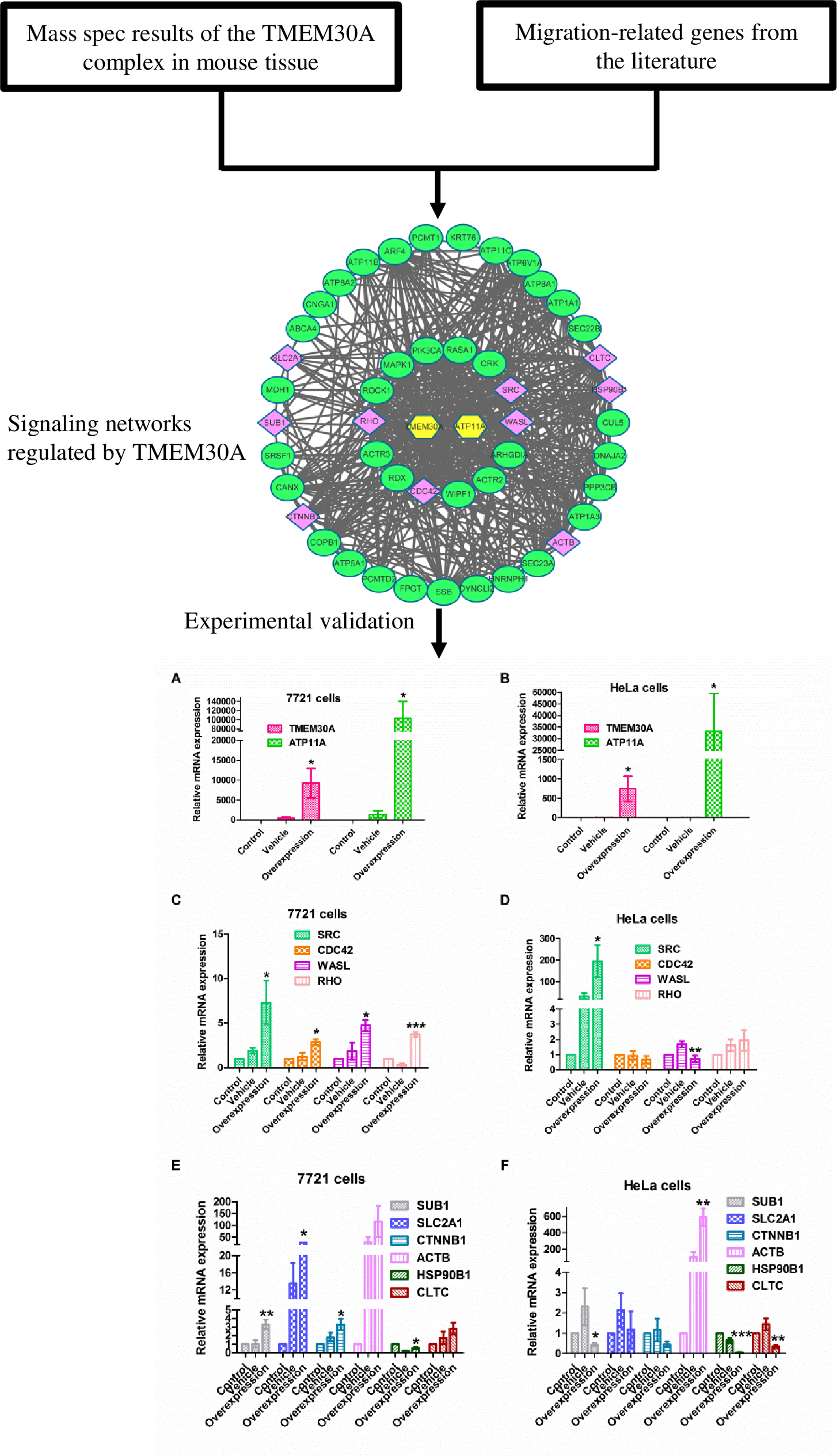

Supplement: S1 Fig — (TIF) [file pone.0179900.s001.tif]

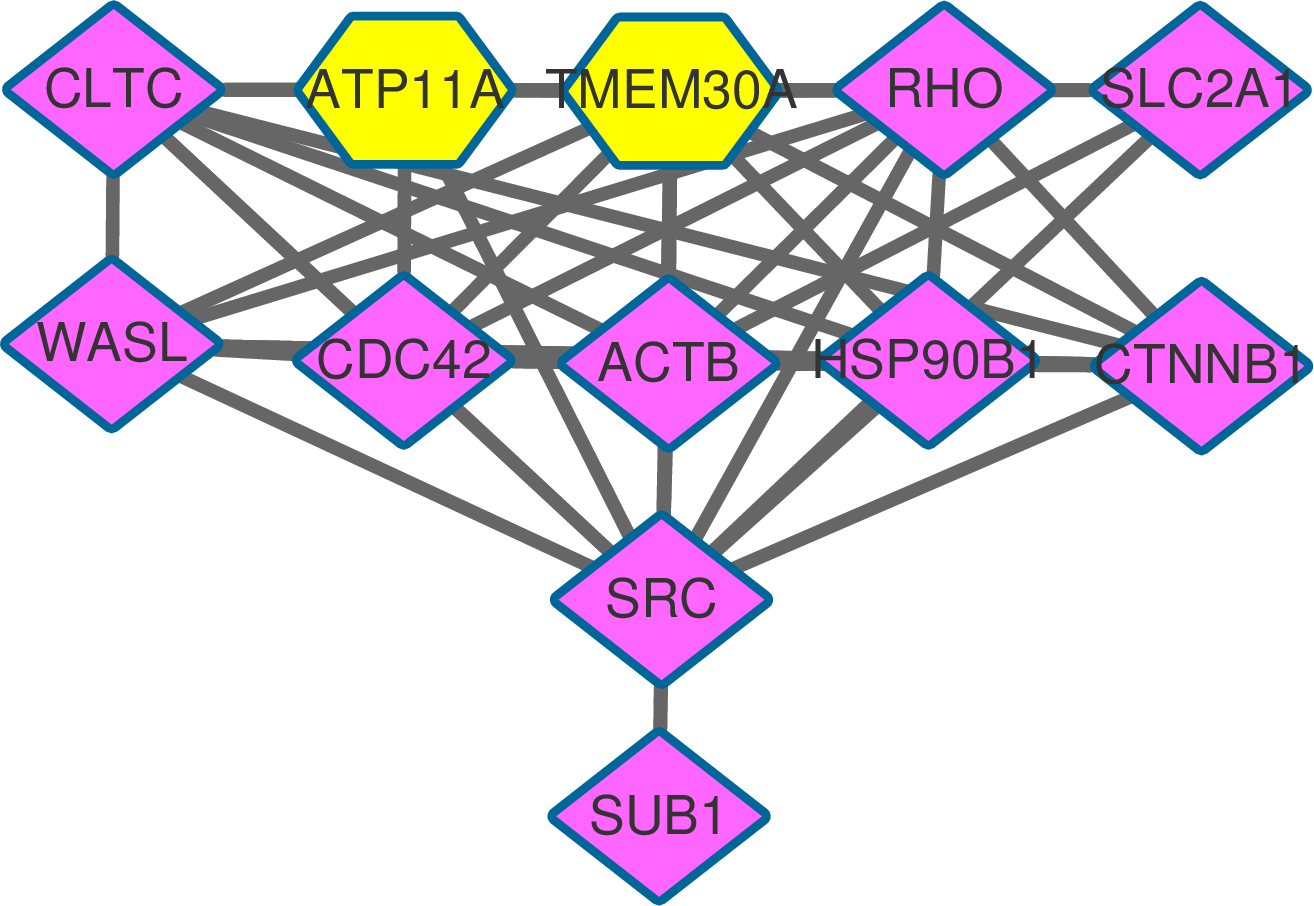

Supplement: S2 Fig — (TIF) [file pone.0179900.s002.tif]
