# Supplementary material for: A biosystems approach to identify the molecular signaling mechanisms of TMEM30A during tumor migration
Source: PLoS One. 2017 Jun 22;12(6):e0179900. doi: 10.1371/journal.pone.0179900 (PMC5481017; doi:10.1371/journal.pone.0179900)
Supplement: S1 File — (PPTX) [file pone.0179900.s006.pptx]

## Slide 1
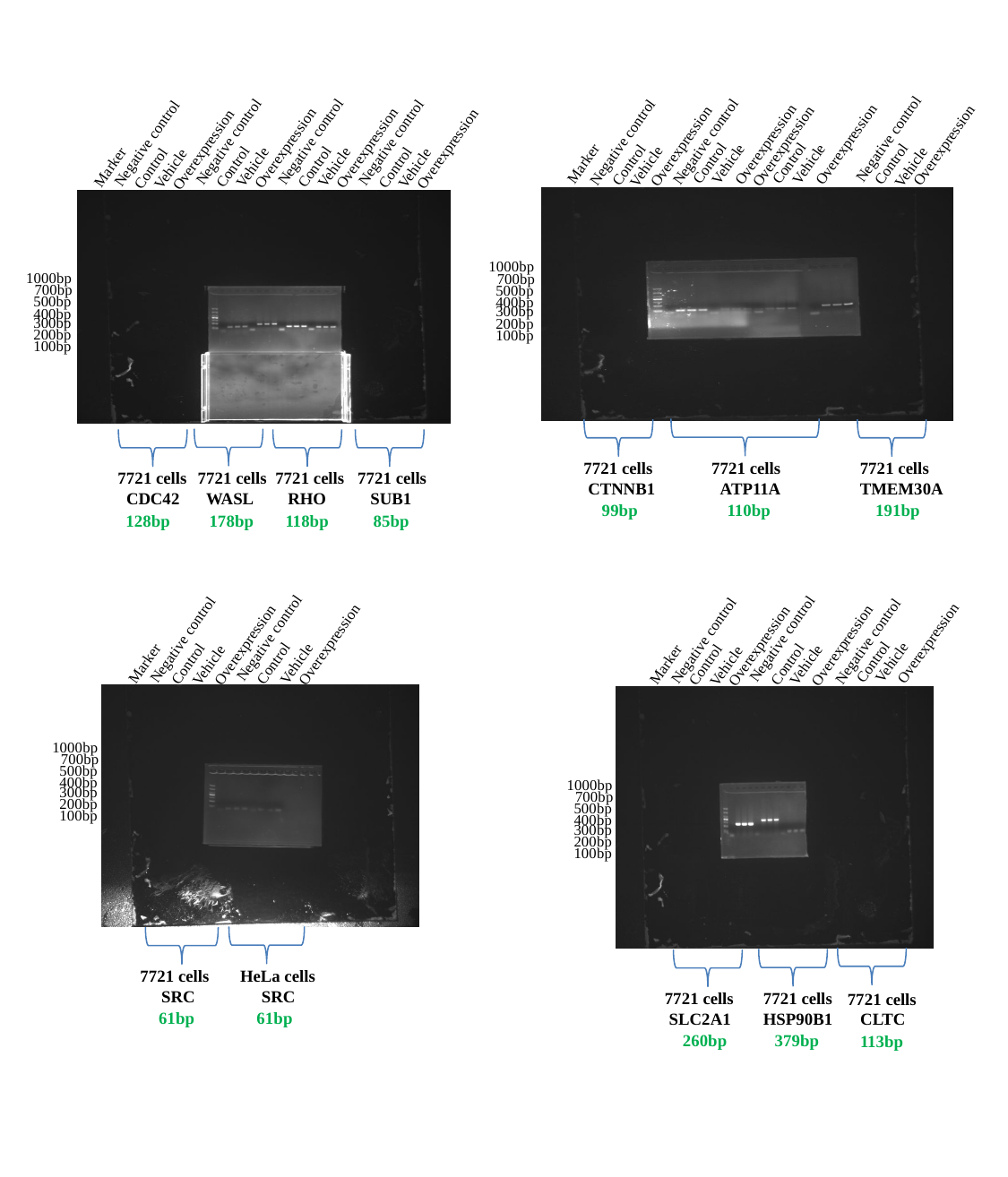

Negative control
Negative control
Negative control
Negative control
Negative control
Negative control
Negative control
Overexpression
Overexpression
Overexpression
Overexpression
Overexpression
Overexpression
Overexpression
Overexpression
Overexpression
Control
Control
Marker
Vehicle
Vehicle
Control
Control
Vehicle
Vehicle
Control
Control
Marker
Vehicle
Vehicle
Control
Vehicle
Control
Vehicle
1000bp
1000bp
 700bp
 700bp
 500bp
 500bp
 400bp
 300bp
 400bp
 300bp
 200bp
 200bp
 100bp
 100bp
7721 cells
 CTNNB1
7721 cells
 ATP11A
7721 cells
TMEM30A
7721 cells
 CDC42
7721 cells
 WASL
7721 cells
 RHO
7721 cells
 SUB1
99bp
110bp
191bp
128bp
178bp
118bp
85bp
Negative control
Negative control
Negative control
Negative control
Negative control
Overexpression
Overexpression
Overexpression
Overexpression
Overexpression
Control
Vehicle
Control
Marker
Control
Vehicle
Control
Marker
Control
Vehicle
Vehicle
Vehicle
1000bp
 700bp
 500bp
 400bp
1000bp
 300bp
 700bp
 200bp
 500bp
 100bp
 400bp
 300bp
 200bp
 100bp
7721 cells
 SRC
HeLa cells
 SRC
7721 cells
 SLC2A1
 7721 cells
 HSP90B1
 7721 cells
 CLTC
61bp
61bp
260bp
379bp
113bp

## Slide 2
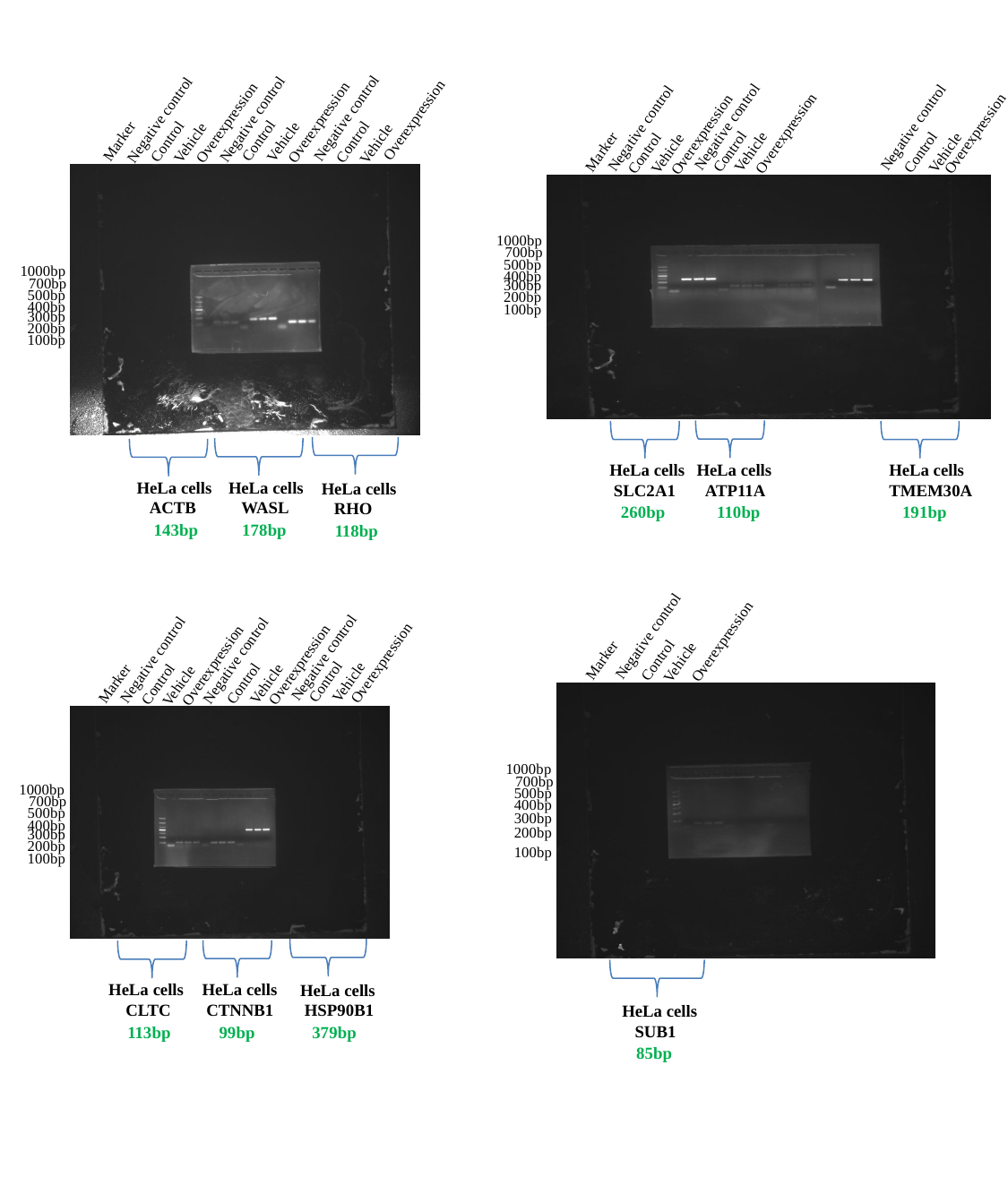

Negative control
Negative control
Negative control
Overexpression
Overexpression
Overexpression
Negative control
Negative control
Negative control
Overexpression
Overexpression
Overexpression
Control
Marker
Control
Vehicle
Control
Vehicle
Vehicle
Control
Marker
Vehicle
Control
Vehicle
Control
Vehicle
1000bp
 700bp
 500bp
1000bp
 400bp
 700bp
 300bp
 500bp
 200bp
 400bp
 100bp
 300bp
 200bp
 100bp
HeLa cells
 SLC2A1
HeLa cells
 ATP11A
HeLa cells
TMEM30A
HeLa cells
 ACTB
 HeLa cells
 WASL
 HeLa cells
 RHO
260bp
110bp
191bp
 143bp
178bp
118bp
Negative control
Overexpression
Negative control
Negative control
Marker
Control
Negative control
Vehicle
Overexpression
Overexpression
Overexpression
Control
Vehicle
Control
Marker
Control
Vehicle
Vehicle
1000bp
 700bp
1000bp
 500bp
 700bp
 400bp
 500bp
 300bp
 400bp
 200bp
 300bp
 200bp
 100bp
 100bp
HeLa cells
 CLTC
 HeLa cells
 CTNNB1
 HeLa cells
 HSP90B1
 HeLa cells
 SUB1
113bp
99bp
379bp
85bp

## Slide 3
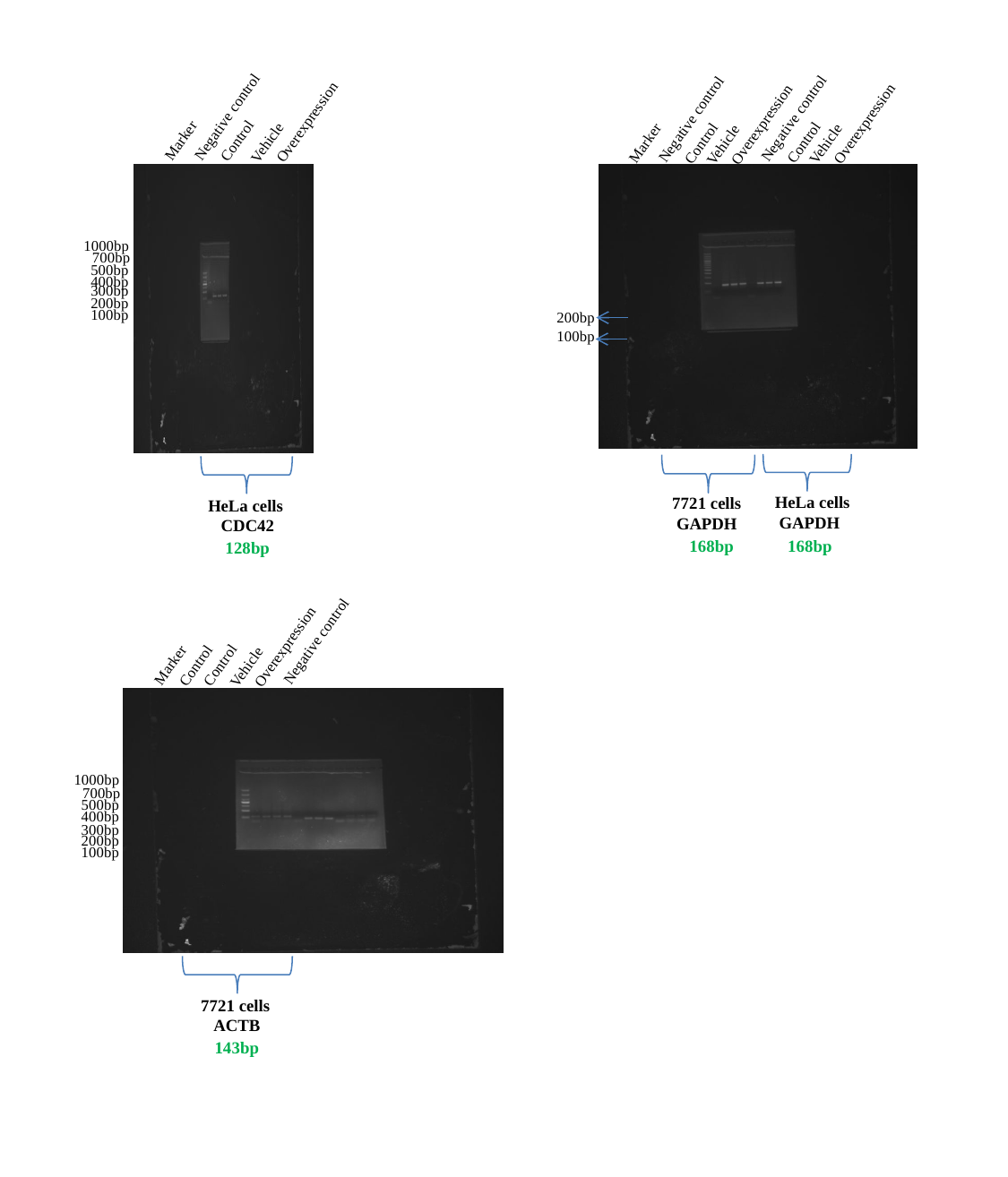

Negative control
Negative control
Negative control
Overexpression
Overexpression
Overexpression
Marker
Control
Control
Vehicle
Marker
Control
Vehicle
Vehicle
1000bp
 700bp
 500bp
 400bp
 300bp
 200bp
 100bp
 200bp
 100bp
HeLa cells
 GAPDH
7721 cells
 GAPDH
HeLa cells
 CDC42
 168bp
168bp
 128bp
Negative control
Overexpression
Marker
Control
Control
Vehicle
1000bp
 700bp
 500bp
 400bp
 300bp
 200bp
 100bp
7721 cells
 ACTB
 143bp
